# Adult attention-deficit hyperactivity disorder: A database analysis of South African private health insurance

**DOI:** 10.4102/sajpsychiatry.v23.1010

**Published:** 2017-01-31

**Authors:** Renata Schoeman, Manie de Klerk

**Affiliations:** 1 Faculty of Medicine and Health Sciences, Department of Psychiatry, Stellenbosch University, South Africa

## Abstract

**Background:**

Adult attention-deficit hyperactivity disorder (ADHD) is a chronic, costly and debilitating disorder. In South Africa (SA), access to funding for care and treatment of ADHD is limited, and research is lacking.

**Aim:**

This study aimed to establish the current situation with regard to the psychiatric management of and funding for treatment of adult ADHD in the private sector in SA.

**Methods:**

A diagnostically refined retrospective claims database analysis was conducted. We examined the prevalence, costs and funding profile of claims over a 2-year period for adult beneficiaries with possible ADHD of a large medical administrator in SA.

**Results:**

The prevalence of adult ADHD was lower than published international rates. The presence of adult ADHD increased the prevalence of comorbidity and doubled the health care costs of beneficiaries. Contrary to public belief, comorbidities (including their medicine costs) rather than psychiatric services or medicines were the main cost drivers.

**Conclusion:**

The current private health insurance funding model for ADHD limits access to funding. This affects early diagnosis and optimal treatment, thereby escalating long-term costs. Improved outcomes are possible if patients suffering from ADHD receive timely and accurate diagnosis, and receive chronic and comprehensive care. Balanced regulation is proposed to minimise the risk to both medical schemes and patients. A collaborative approach between stakeholders is needed to develop an alternative cost-effective funding model to improve access to treatment and quality of life for adults with ADHD in SA.

## Introduction

Attention-deficit hyperactivity disorder (ADHD) has received increased scientific, clinical and public attention over the past few decades. ADHD is the most common psychiatric disorder in children, affecting 2.0% to 16.0% of the school-age population.^1^ It is now widely accepted that an estimated 60.0% to 70.0% of patients’ symptoms persist into adulthood, with estimates of the prevalence of adult ADHD between 2.5% and 4.3%.^2^

ADHD is a costly, chronic disorder, with significant impact on the quality of life (QOL) of patients and their families. The burden of disease (BOD) is significant, with the disability-adjusted life years (DALYs) calculated as 424 per 100 000.^3^ Comorbidity, estimated at more than 50% with ADHD, contributes to the BOD and reduced QOL of patients with ADHD.^4,5^

In an analysis of all medical, pharmaceutical and disability claims in an administrative database (*N* > 100 000), resource utilisation of individuals with ADHD and their family members was contrasted with a matched control sample of patients without ADHD. The direct costs of ADHD in terms of annual average expenditure per patient, outpatient costs, inpatient costs and prescription drug costs were two- to threefold the costs of matched controls.^6^

ADHD causes significant personal, interpersonal and social burden, impacting negatively on overall QOL. Many studies have confirmed the efficacy and effectiveness of both stimulant and non-stimulant medication in the treatment of ADHD in children, adolescents and adults. Although pharmacotherapy plays a primary role in the treatment of ADHD, psychosocial interventions (psycho-education, cognitive behavioural therapy, supportive coaching or assistance with daily activities) are an integral part of management.^7,8,9,10,11^

Despite the known efficacy of treatment and the substantial costs of untreated ADHD, access to health care and treatment is not a given for many patients in emerging markets. This holds true for SA where research indicates poor identification and treatment of common mental disorders at primary health care level and limited access to specialist resources with a service delivery and treatment gap of up to 75%.^12,13^ Medication options are often limited in emerging markets, and in SA, psychiatrists and patients do not have access to the medicines available in established markets.

The lifetime prevalence of ADHD in SA is unknown. Extrapolating the known international prevalence information to the South African context, the expected number of adults aged between 20 and 50 affected by ADHD would be between 771 264 (3%) and 1 285 439 (5%).

In SA, funding for treatment for children with ADHD is private, either via medical schemes or via the state sector (limited). However, adults with ADHD have even less access to care. Some medical schemes that cover for childhood ADHD often do not provide benefits for the treatment of adult ADHD, and patients can often not afford private treatment in addition to their monthly contributions to these funds.

Our study, the first in the field in SA, aimed to establish the current situation with regard to the psychiatric management of and funding for treatment of adult ADHD in the private sector as the basis for a proposal for a new funding model in order to improve access to treatment and QOL for adults with ADHD in emerging markets such as SA.

## Methods

A triangulated study was conducted consisting of a retrospective claims database analysis, a survey and in-depth interviews. In this article, we report on findings on the quantitative analysis of a retrospective claims database using medical data, pharmacy data and enrolment information as captured for the largest administrator of medical schemes in SA, representing 3 million beneficiaries (29% of all beneficiaries across 17 medical schemes).

### Inclusion criteria

To be included, claims submitted to the medical scheme had to be:

- for adult beneficiaries (aged 18–60)
- who had one or more outpatient medical claims for ADHD as indicated by relevant ICD-10 codes between 1 July 2011 and 30 June 2013^14^
- who had received scripts or claimed for methylphenidate (MPH) derivatives, atomoxetine or bupropion as indicated by relevant National Pharmaceutical Product Index (NAPPI) codes.^15^

In SA, only MPH derivatives and atomoxetine are registered for the treatment of ADHD. International guidelines also include, amongst others, buproprion – which is used off-label for the treatment of ADHD in SA – especially in adults who tolerate the other registered drugs poorly. Although other drugs such as clonidine, guanfacine, tricyclic antidepressants, modafanil and venlafaxine are mentioned in the guidelines as third-line treatment for ADHD, none of these are indicated for this specific use in SA and are therefore excluded from this study. Concerns about diversion of medication (for recreational purposes or ‘cognitive enhancement’) and non-compliance are relevant, but impossible to ascertain. For the purposes of this study, it was assumed that patients who fill their scripts at the pharmacy are indeed compliant with the use thereof.

### Data collection

Medical data, pharmacy data and enrolment information as captured for a large medical scheme administrator during the study period were analysed. Data were collected during September 2013 as all delayed claims were captured by then.

### Data analysis

Descriptive statistics and correlations were used to describe the patient profile, disease profile, practitioner profile, treatment profile and funding profile. All analyses were conducted with STATISTICA, Version 12 and Microsoft Office Excel 2013.

### Ethical considerations

Approval for the project was obtained from the University of Stellenbosch Business school ethics committee.

## Results

Note: Results in the text are reported as mean ± sd.

### General

For the period under investigation, the total number of beneficiaries managed by the medical scheme administrator (i.e. potential claimants) in the age group under investigation was 1 390 654. To be included in the sample, claims had to be linked with either an applicable ICD or NAPPI code (see Inclusion criteria section). In this study, the total number of claims (i.e. individual data points collected) for the 2-year period analysed totalled 1 740 751, originating from 15 934 beneficiaries. These claims totalled R812 916 038.20, amounting to 1.91% of the total value (R42 670 01 47 96) of claims.

Duplicates (beneficiaries who migrated between options within a specific medical scheme) were merged for a total number of 15 203 who fulfilled the inclusion criteria (i.e. a prevalence rate of possible ADHD of 1.09% – calculated as 1 390 654/15 203). The mean age of beneficiaries in the study sample was 33.83 ± 12.64 years at the time the first claim for the beneficiary was captured. The mean number of claims per beneficiary was 115 ± 138, with a median of 138 claims.

**FIGURE 1:**
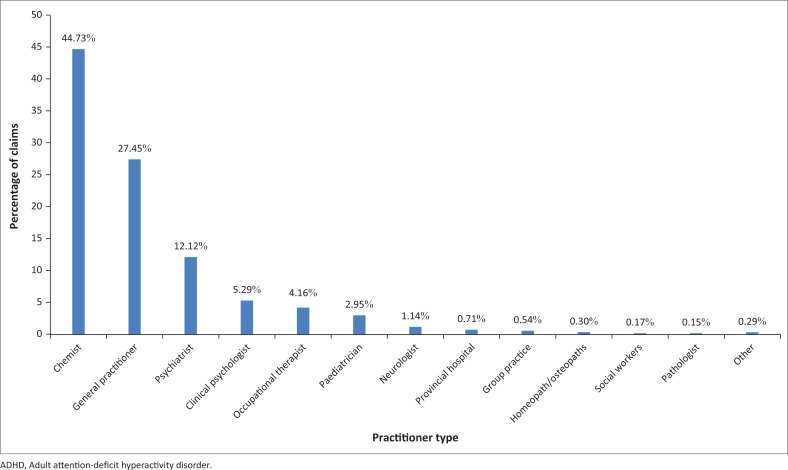
Percentage of claims linked to ADHD diagnostic codes.

The largest number and value of claims were generated by pharmacies (*n* = 620 574, 35.65%). This is to be expected as almost every other service contact (e.g. for comorbid conditions) will generate a script for medication. This also reflects the issuing of chronic medication or repeat scripts without another service contact. The second-most claims were generated by general practitioners (GPs) (*n* = 225 142, 12.93%) – the first point of health care service contact for most patients and for most conditions. Private hospitals and pathologists followed closely with 224 496 (12.90%) and 205 921 (11.83%) claims, respectively. Psychiatrists’ claims contributed to only 2.7% of claims (*n* = 46 974), whereas psychiatric clinics generated 0.20% of claims (*n* = 3490). Other service providers each contributed to less than 0.3% of the total claims (see Figure 1).

### Attention-deficit hyperactivity disorder

For a more accurate estimate of prevalence and costs of ADHD, claim history was cross tabulated according to relevant ICD codes and NAPPI codes (see Tables 1 and 2).

**TABLE 1:** The prevalence of adult ADHD according to claim history.

NAPPI codes	ICD-10 codes		Total
	‘ADHD’	Other	
ADHD	4291 (0.25%)	48 941 (2.81%)	53 232 (3.06%)
Other	6257 (0.36%)	1 681 262 (96.58%)	1 687 519 (96.94%)
**Total**	**10 548 (0.61%)**	**1 730 203 (99.39%)**	**1 740 751 (100.00%)**

ADHD, Adult attention-deficit hyperactivity disorder; NAPPI, National Pharmaceutical Product Index.

**TABLE 2:** The cost of adult ADHD according to claim history.

NAPPI codes	ICD-10 codes		Total
	ADHD	Other	
ADHD	R458 14 82 (0.56%)	R399 21 883 (4.91%)	R445 03 365 (5.47%)
Other	R358 12 11 (0.44%)	R764 83 1462 (94.08%)	R768 41 2673 (94.53%)
**Total**	**R816 26 93 (1.00%)**	**R804 75 3345 (99.00%)**	**R812 91 6038 (10.00%)**

ADHD, Adult attention-deficit hyperactivity disorder; NAPPI, National Pharmaceutical Product Index.

Amongst the beneficiaries included, the majority of claims (1 681 262, 96.58%) were not linked to the relevant codes. This contributed to 94.08% of the total value of claims. This may be because of the claim being unrelated to ADHD, poor coding by service providers or the condition not being seen as the primary reason for the service contact (e.g. therefore not indicated during submission of claims or on scripts). Some of these claims might in fact still have been associated with the presence of ADHD.

Of the 10 548 (0.61%) claims linked to ICD codes for ADHD, 44.73% were generated by pharmacies, followed by GPs (27.45%) and psychiatrists (12.12%). Although pharmacies submitted most claims linked to the diagnostic code, these reflect scripts issued according to the diagnostic codes as documented by other service providers (e.g. GPs and psychiatrists) and are not reflective of diagnosis *per se*. Furthermore, 6257 (0.35%) claims were associated with NAPPI codes unrelated to ADHD. This contributed to 0.44% of the total value of claims. Medical practitioners are not consistent in documenting the F-codes on scripts. The majority of the codes linked to psychiatrists and GPs will be reflective of consultations linked to the diagnosis (primary or secondary diagnoses), whereas some of the claims may also be linked to medication dispensation. These indicate claims that originated because of comorbid conditions (i.e. the ADHD was not the focus of attention) or claims where ADHD received non-conventional treatment (e.g. medication which was used off-label).

Of the 53 232 (3.06%) claims linked to NAPPI codes (i.e. medication dispensed) for ADHD, the majority were generated by pharmacies (*n* = 5172, 97.14%), whereas private hospitals dispensed 1.62% (*n* = 863) and GPs 0.98% (*n* = 472). Of these claims, 48 941 claims (2.81%) were linked to ICD codes unrelated to ADHD, contributing 4.91% to the total value of claims. This could be an indication of off-label use of ADHD medication (e.g. as cognitive enhancement in patients who do not have ADHD or as augmentation in conditions such as treatment resistant depression) or poor script habits of service providers (e.g. where the diagnostic codes are not indicated on the scripts).

Our ‘best estimate’ of the prevalence of ‘true’ adult ADHD according to claim history would, therefore, be those claims that captured both relevant ICD codes and relevant NAPPI codes. These combined codes represented 4291 (0.25%) claims included in the study sample amounting to 0.56% of the total value of claims in the study sample. These direct costs are attributable to medication costs (R1 762 954; 38.48%), psychiatric services (R14 98 14; 3.27%), GP consultation (R2 668 713; 58.25%) and other.

### Comorbidity

In the study sample, 3106 (20.43%) of the 15 203 beneficiaries had non-psychiatric comorbid disorders (i.e. an ICD code other than psychiatric F-codes) (see Figure 1). Respiratory disorders were the most prevalent comorbid condition, followed by haematological and immunological disorders. Of the total claims, 5163 (0.30%) claims were linked to an ICD F-code (i.e. a psychiatric diagnosis) in addition to the F90 code of inclusion (i.e. ADHDs). The most common psychiatric comorbidities were adjustment disorders (*n* = 409, 29.5%, 2.7% of the sample), non-organic sleep disorders (*n* = 269, 19.4%, 1.8%), anxiety disorders (*n* = 181, 13.1%, 1.2%), mood disorders (*n* = 191, 13.8%, 1.2%) and substance-related disorders (*n* = 32, 2.3%, 0.2%).

### Treatment profile

The single biggest contributor to the number of generated claims was medicines related (*n* = 763 892, 38.48%) of which 318 039 (16.00%) claims were for chronic medication and 355 313 (18.00%) claims for acute medication. Chronic medication refers to all medication obtainable by a repeat script – which would include atomoxetine and bupropion. Acute medication refers to all medications obtained on an acute script. MPH derivatives, although used as chronic medicine, were therefore included as acute medication because monthly scripts are needed for schedule 6 medication. When a patient’s chronic benefits are depleted, medication claims will also be submitted as acute medication claims.

ADHD medication amounted to 5.47% of costs in the study sample. When examining the relevant NAPPI codes in the study sample, it is clear that the majority of claims (*n* = 30 582, 57.45%) were for bupropion. The second most common drug prescribed for treatment of ADHD in the study sample was osmotic-controlled-release oral delivery system (OROS^®^) MPH (*n* = 10 128, 19.03%), followed by IR MPH (*n* = 6230, 11.70%) and MPH LA (*n* = 5662, 10.64%). Atomoxetine lagged behind the other scripts (*n* = 630, 1.18%).

Psychiatric claims contributed to a total of 3.27% of the ADHD-related claims. Of these, 36.48% consisted of consultations, 51.02% of individual psychotherapeutic sessions, 1.69% of group therapy and 2.13% of electroconvulsive therapy. The writing of out-of-consultation scripts contributed to 8.68% of psychiatric claims. Follow-up consultations (brief, i.e. less than 20 minutes, and longer, i.e. 21 to 40 minutes) consisting of psychotherapeutic interventions were amongst the top 25 claim drivers, albeit the contribution, relative to other claim drivers, was relatively small.

Although psychiatric consultations consisting of psychotherapeutic interventions were amongst the top 25 utilised services (in terms of number of claims generated), the contribution to cost, relative to other utilised services, was relatively small and not that impactful.

### Funding profile

The mean cost per claim (MCPC), the mean benefit per claim (MBPC) – which reflect the cost to scheme, and the mean cost to patient (MCTP) for each patient were calculated. The MCPC, the MBPC and the MCTP for each medical scheme option (*n* = 38), as well as the total cost of claims (TCC), the total benefits paid and the total cost to patients are summarised in Table 2.

Of the TCC, medical schemes paid 91.95% in benefits, whereas beneficiaries in the study sample were responsible for 8.05% of the costs.

## Discussion

### Prevalence of adult ADHD

The prevalence of adult ADHD in our sample was estimated at 1.09%. This is clearly lower than the population estimates for adult ADHD of 2 to 5% as per previous studies.^2,16^

Although adult ADHD is established as a recognised disorder abroad, in SA the diagnosis of ADHD is hampered by a lack of awareness of the disorder, non-recognition of the disorder and a lack of access to diagnosis.^17^ It is therefore possible that beneficiaries in the database analysed have undiagnosed ADHD, or untreated ADHD. A lack of funding also hampers access to treatment, and these patients will, therefore, not be identified in the study sample because of the fact that their condition may not have attracted claims.

The results of this study are also strongly reliant on the coding accuracy of service providers (ICD codes) and the administrative accuracy of pharmacies (NAPPI codes). Furthermore, the study sample was limited to restricted schemes only and may not be generalisable to open medical schemes.

### The costs of adult ADHD

As per Results section (General), the total value of claims generated for the patients in the study sample contributed 1.9% to the total value of claims for this age group. Considering our sample size, the presence of possible ADHD virtually doubled the costs (i.e. total value of claims for all reasons) per claiming beneficiary. This is in accordance with the previous studies which found that direct costs in patients with ADHD amounted to up to three times the costs in matched controls.^5,6^

As per Results section (Attention-deficit hyperactivity disorder), only 0.01% of the total value of all claims could be attributed to ‘true’ adult ADHD. The direct cost of treating ADHD *per se* is, therefore, far less than the cost of treatment of comorbid conditions (0.44% of the value of claims [R3581211]), as well as the use of ADHD medication for purposes other than ‘true’ ADHD (4.91% of the value of claims [R39921883]). The total cost of ADHD medication cost in the study sample (5.47% of costs) is comparable with the findings of Birnbaum et al., who calculated medication costs for treatment of ADHD as 7% of all direct and indirect costs.^18^

Although a comprehensive, multi-modal, management approach for adults with ADHD is recommended, the majority of services claimed for were medication related. Only 1% of the claims generated were for services rendered by psychologists. Claims for individual psychotherapy also amounted to 1% of claims. If we consider claims originating from psychiatrists for ADHD *per se,* it appears that psychiatrists do emphasise the need for psychotherapy (see Results section: Treatment profile). Although psychiatric consultations consisting of psychotherapeutic interventions were amongst the top 25 utilised services (in terms of number of claims generated), the contribution to cost, relative to other utilised services, was relatively small and not that impactful.

Comorbidity of disease and complications of psychiatric disorders are important cost drivers. Psychiatric comorbidity in our study was more prevalent than in the general psychiatric population for both anxiety disorders (13.1% vs. 8.1%) and mood disorders (13.8% vs. 4.5%).^19^ Although substance disorders were less prevalent in the study sample than in the general South African population (2.3% vs. 5.8%), this is most likely an underestimation as patients often do not disclose the use or misuse of substances, and service providers also, even if aware of the presence thereof, seldom code it when submitting claims.

Multiple comorbidities were also more prevalent in the study database sample than in the general population with 9.09% having had one additional psychiatric diagnosis (vs. 3.9%), while 10.95% had two or more psychiatric diagnoses (vs. 1.4%). However, comorbidity in this study was still lower than previous estimations in excess of 50% reported elsewhere.^4,5^ This may once again reflect access to care and diagnosis.

Psychiatric services *per se* are not the main utilised service, nor the main cost driver in the study sample, despite the prevalence of psychiatric disorders. This may be indicative of a lack of access for patients to (proper) diagnosis and treatment with regard to ADHD. This is a concern with regard to the use of scheduled medication such as stimulants, where in the study sample it would be expected that patients at least had six-monthly (or at the least yearly) reviews should they suffer from ADHD. Within psychiatry, consultations remain the largest claim driver, but the biggest overall cost driver remains medication. It is difficult to determine whether warranted utilisation versus the unregulated or injudicious use of medications registered for the use in ADHD may be a significant cost driver. Areas where costs can potentially be contained would be medication, hospitalisations and special examinations.

### The funding of adult ADHD

Of the TCC, medical schemes paid 91.95% in benefits, whereas beneficiaries in the study sample were responsible for 8.05% of the costs. Because of the multiple medical schemes and options included in the researcher’s analysis, the study did not distinguish between specific services and medication paid from chronic benefits, savings accounts or as out of pocket (OOP) expense to beneficiaries. This is not in agreement with findings from a national survey amongst psychiatrists which indicated a heavier burden on patients with 35.98% of the costs of psychiatric consultations, 41.17% of medication for the treatment of ADHD, 62.20% of supportive and alternative therapies, occurring as OOP expenses.^17^

## Conclusion

Adult ADHD is a chronic, costly and debilitating disorder. This study, the first in the field of adult ADHD in SA, aimed to establish the current situation in SA with regard to the psychiatric management of and funding for treatment of adult ADHD in the private sector.

Lower than international prevalence rates may reflect a lack of awareness of the disorder, lack of access to diagnosis and treatment, and poor coding habits of health care practitioners. The cost drivers in this study were not psychiatric services or medication, but the presence of comorbid conditions, as well as use of medication for non-ADHD-related reasons.

The current medical scheme funding model for ADHD limits access to diagnosis and optimal treatment, thereby escalating long-term direct, indirect and intangible costs and the BOD on the patients, their families and society. Improved outcomes are possible if patients suffering from ADHD receive a timely and accurate diagnosis and receive comprehensive care – which would include psychopharmacological interventions, behavioural interventions and support.

It is proposed that schemes should recognise adult ADHD as a chronic disorder, which needs chronic treatment and, therefore, remunerate for services and medication from chronic benefits. To decrease the risk to medical schemes, balanced regulation is suggested. The bar should be raised in terms of receiving the diagnoses of adult ADHD, for example, through partnerships with psychiatrists or centres of excellence where comprehensive assessment is available to ensure that the threshold to obtaining the diagnosis is sensitive and specific. Guidelines should be established with regard to the diagnostic process, relevant special examinations, as well as recommended treatment regimes. Once the diagnosis is confirmed, a patient should have access to comprehensive treatment. Once stabilised, follow-up can take place at primary health care level, leading to further cost savings through use of relevant resources. A collaborative approach between stakeholders with public-private partnerships is crucial in this process of addressing research (e.g. community-based naturalistic studies, clinical trials and health economic studies), education (training and continued medical education), information and communication (e.g. through national awareness campaigns) and improving practices (e.g. with regard to coding practices, diagnosis and treatment). The aim is to break down the barriers with regard to a lack of knowledge and a lack of funding in order to provide access to care and treatment and to develop an alternative cost-effective funding model for the treatment of adult ADHD in SA.
